# Supplementary material for: Rpd3L and Hda1 histone deacetylases facilitate repair of broken forks by promoting sister chromatid cohesion
Source: Nat Commun. 2019 Nov 15;10:5178. doi: 10.1038/s41467-019-13210-5 (PMC6858524; doi:10.1038/s41467-019-13210-5)
Supplement: Supplementary file 1 — Supplementary Information [file 41467_2019_13210_MOESM1_ESM.pdf]

SUPPLEMENTARY INFORMATION

Rpd3L and Hda1 histone deacetylases facilitate repair of broken forks by promoting sister chromatid cohesion.

Ortega *et al.*

SUPPLEMENTARY FIGURES

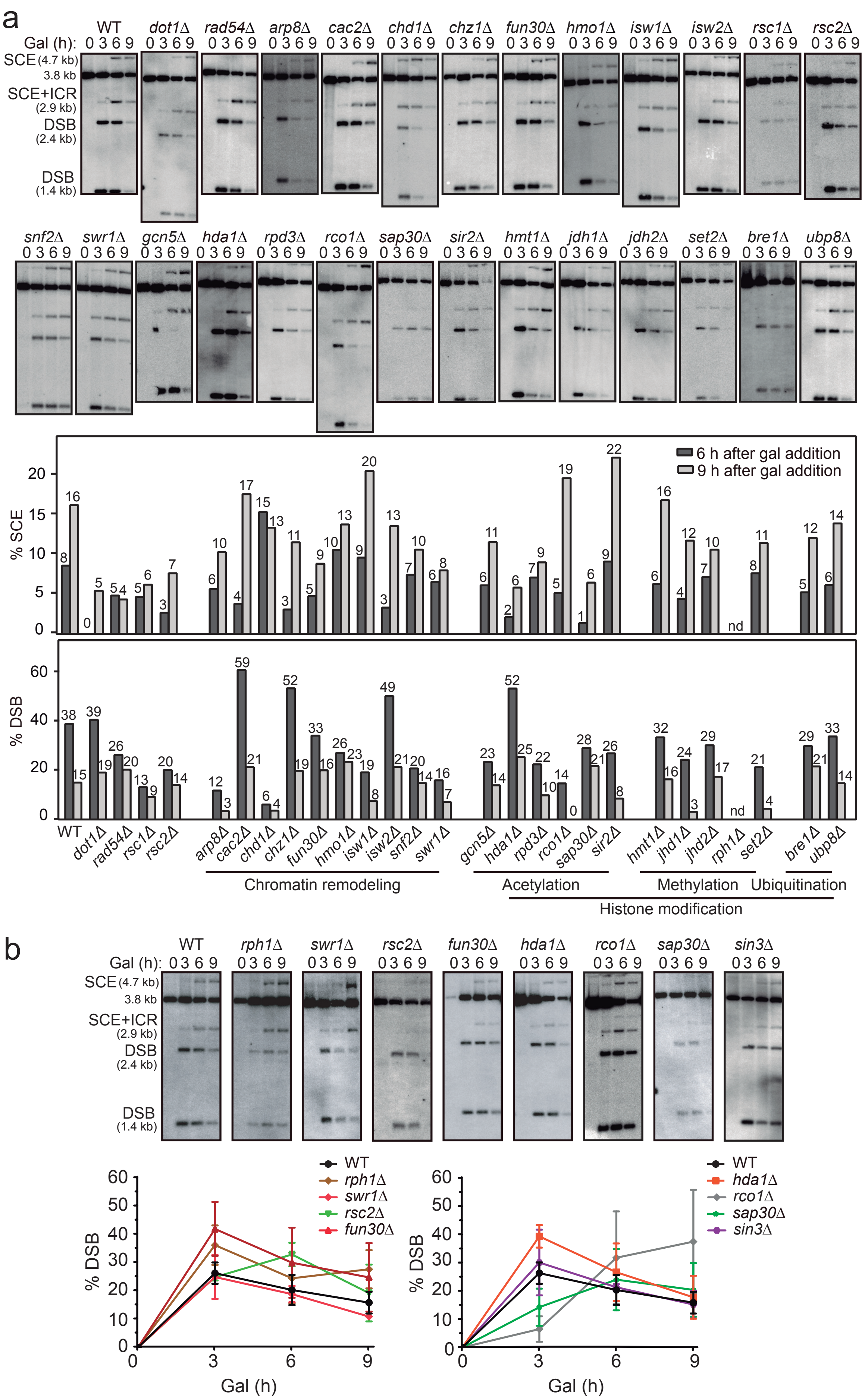

Supplementary Figure 1. Screening for factors involved in SCR among chromatin remodeling and histone modifiers. Related to Figure 1.
(A) Representative Southern blots and quantification of the DSB 2.4 and 1.4 -Kb detected after 9 hours of HO induction in wild-type (BY4741) and the indicated mutant strains from the Euroscarf collection (see Table S1) transformed with the pTHGH plasmid that contains both the TINV-HO system and GAL::HO. (n = 1).
(B) Representative Southern blots and analyses of the 2.4 and 1.4-Kb DSB fragments during a time-course experiment after HO induction in wild-type (WSR-7D), r*sc2*∆ (WSRSC2), *fun30*∆ (WSFUN30), *rph1*∆ (WSRPH1), *swr1*∆ (WSSWR1), *hda1*∆ (WSHDA1), *rco1*∆ (WSRCO1), *sap30*∆ (WSSAP30) and *sin3*∆ (WSSIN3) strains transformed with pRS316-TINV (n ≥ 2).
Means and SEM are plotted in all panels. Data underlying this Figure are provided as Source Data file.

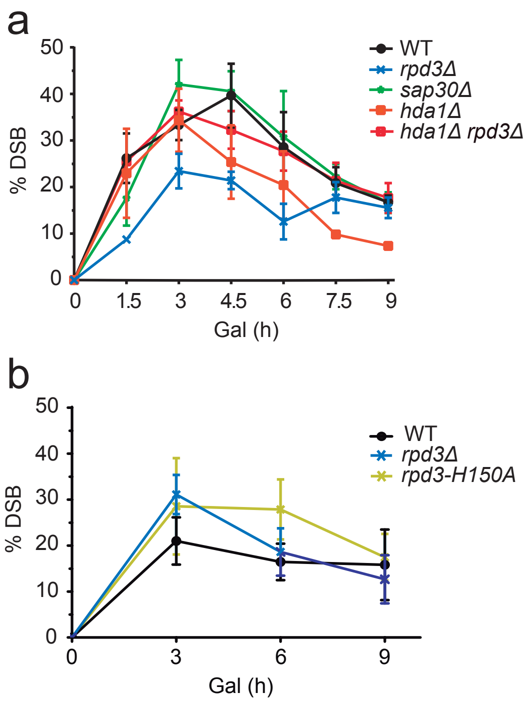

Supplementary Figure 2. Analysis of HO-induced DSB and spontaneous recombination levels in *rpd3*∆ and *sap30*∆ mutants. Related to Figure 3.
(A) Analysis of the 2.4 and 1.4-Kb DSB fragments during a time-course experiment after HO induction in wild-type (WSR-7D), *rpd3*∆ (WSRPD3), *sap30*∆ (WSSAP30), *hda1*∆ (WSHDA1) and *hda1*∆ *rpd3*∆ (WSH1R3) strains transformed with pRS316-TINV (n ≥ 3).
(B) Analysis of the 2.4 and 1.4-Kb DSB fragments during a time-course experiment after HO induction in the WSRPD3 (*rpd3*∆) strain transformed with YEplac112 (*rpd3*∆), YEplac112-Rpd3 (WT) or YEplac112-H150A (r*pd3-H150A*) plasmids (n ≥ 3).
Means and SEM are plotted in all panels. Data underlying this Figure are provided as Source Data file.

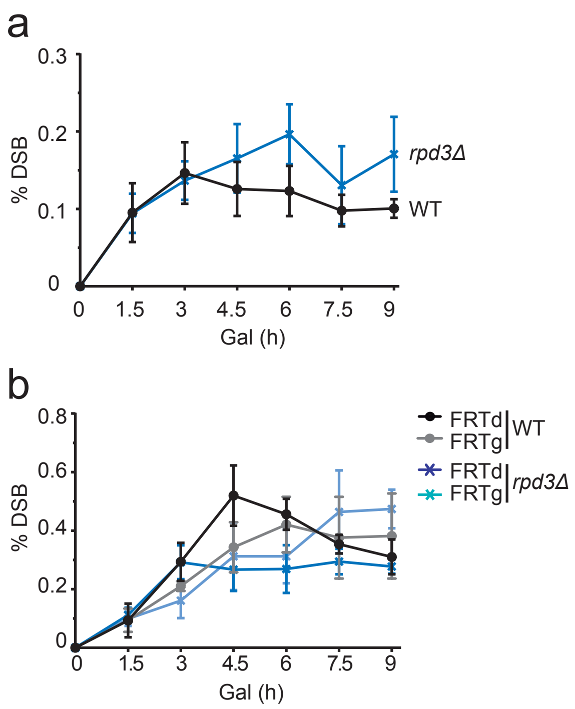

Supplementary Figure 3. Analysis of FLPm-induced DSB using pTINV-FRT, pTINV-FRTd and pTINV-FRTg. Related to Figures 4 and 5.
(A) Analysis of the 2.4 and 1.4-Kb DSB fragments during a time-course experiment after FLPm induction performed at 37ºC in wild-type (WFLP) and *rpd3*Δ (WFRPD3) strains transformed with pTINV-FRT (n ≥ 3).
(B) Analysis of the 2.4 and 1.4-Kb DSB fragments during a time-course experiment after FLPm induction in wild-type (WFLP) and *rpd3*Δ (WFRPD3) strains transformed with pTINV-FRTd or pTINV-FRTg (n = 3).
Means and SEM are plotted in all panels. Data underlying this Figure are provided as Source Data file.

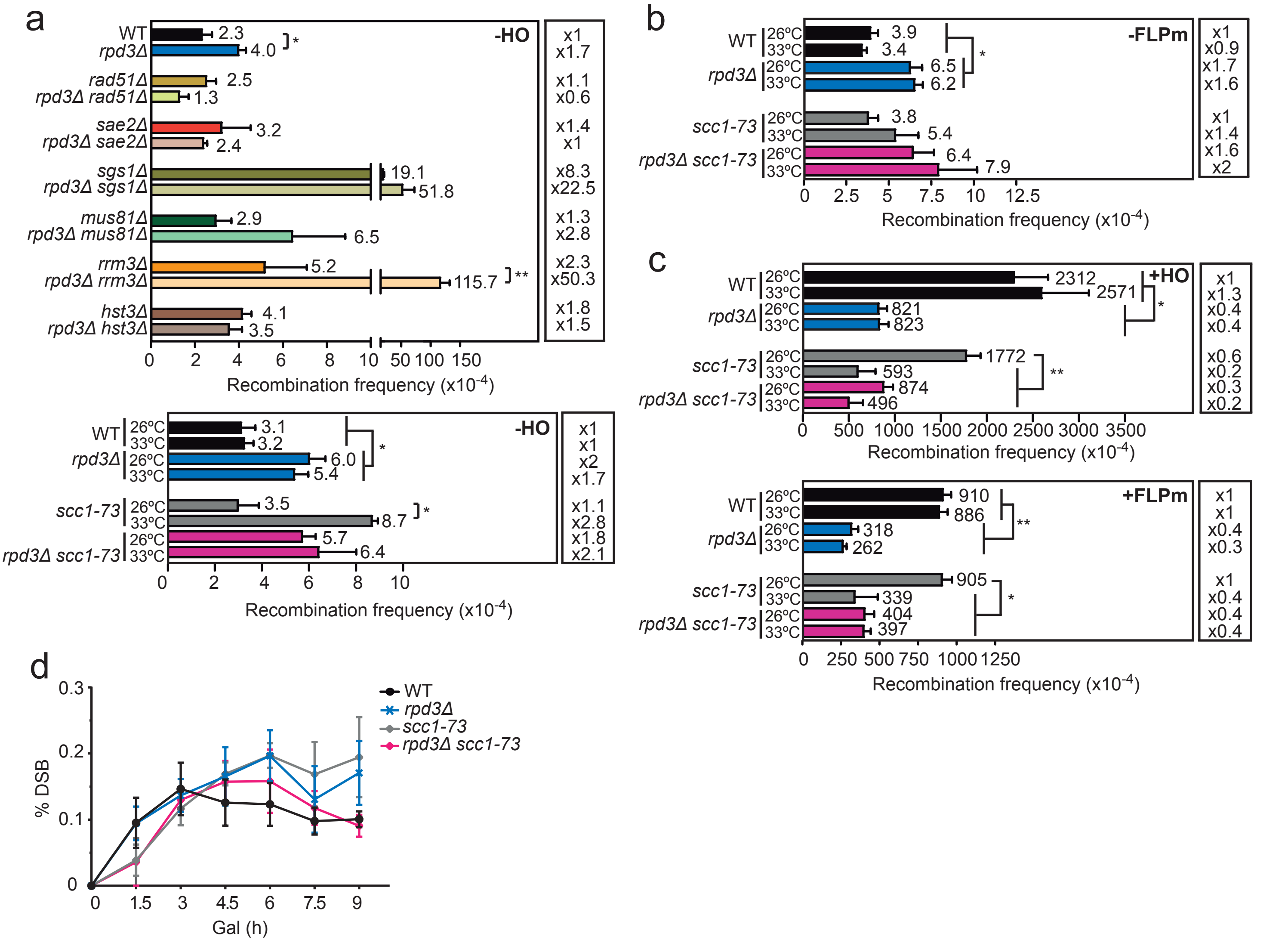

Supplementary Figure 4. Analysis of HO-induced DSB and spontaneous recombination levels in *rpd3*Δ in combination with other SCR-affected mutants. Related to Figure 6.

(A) Analysis of spontaneous (-HO) recombination frequencies at 30ºC (upper panel) and at 26 or 33 ºC (lower panel) in wild-type (WSR-7D), *rpd3*Δ(WSRPD3b), *rad51*Δ (WSRAD51), *rpd3*Δ *rad51*Δ (WSR3R5), *sae2*Δ (WSSAE2), *rpd3*Δ *sae2*Δ (WSR3S2), *sgs1*Δ (WSSGS1), *rpd3*Δ *sgs1*Δ (WSR3SG), *mus81*Δ (WSMUS81), *rpd3*Δ *mus81*Δ (WSR3M8), *rrm3*Δ (WSRRM3), *rpd3*Δ *rrm3*Δ (WSR3RR), *hst3*Δ (WSHST3), *rpd3*Δ *hst3*Δ (WSR3H3), *scc1-73* (WSSCC1) and *rpd3*Δ *scc1-73* (WSR3S1) strains transformed with pRS316-TINV (n ≥ 3).

(B) Analysis of spontaneous (-FLPm) recombination frequencies at 26ºC or 33ºC in wild-type (WFLP), *rpd3*Δ (WFRPD3), *scc1-73* (WFSCC1) and *rpd3*Δ *scc1-73* (WFR3S1) strains transformed with pTINV-FRT (n = 3).

(C) Analysis of HO and FLPm-induced recombination frequencies at 26ºC or 33ºC in wild-type (WS) or wild-type (WFLP), *rpd3*Δ (WSRPD3) or *rpd3*Δ (WFRPD3), *scc1-73* (WSSCC1) or *scc1-73* (WFSCC1) and *rpd3*Δ *scc1-73* (WSR3S1) or *rpd3*Δ *scc1-73* (WFR3S1) strains transformed with pRS316-TINV or pTINV-FRT (n = 3).

(D) Analysis of the 2.4 and 1.4-Kb DSB fragments during a time-course experiment after FLPm induction performed at 37ºC in wild-type (WFLP), *rpd3*Δ (WFRPD3), *scc1-73* (WFSCC1), and *rpd3*Δ *scc1-73* (WFR3S1) strains transformed with pTINV-FRT (n ≥ 3).

Means and SEM are plotted in all panels. *p % 0.05; **p % 0.01 (two-tailed Student's t test). Data underlying this Figure are provided as Source Data file.

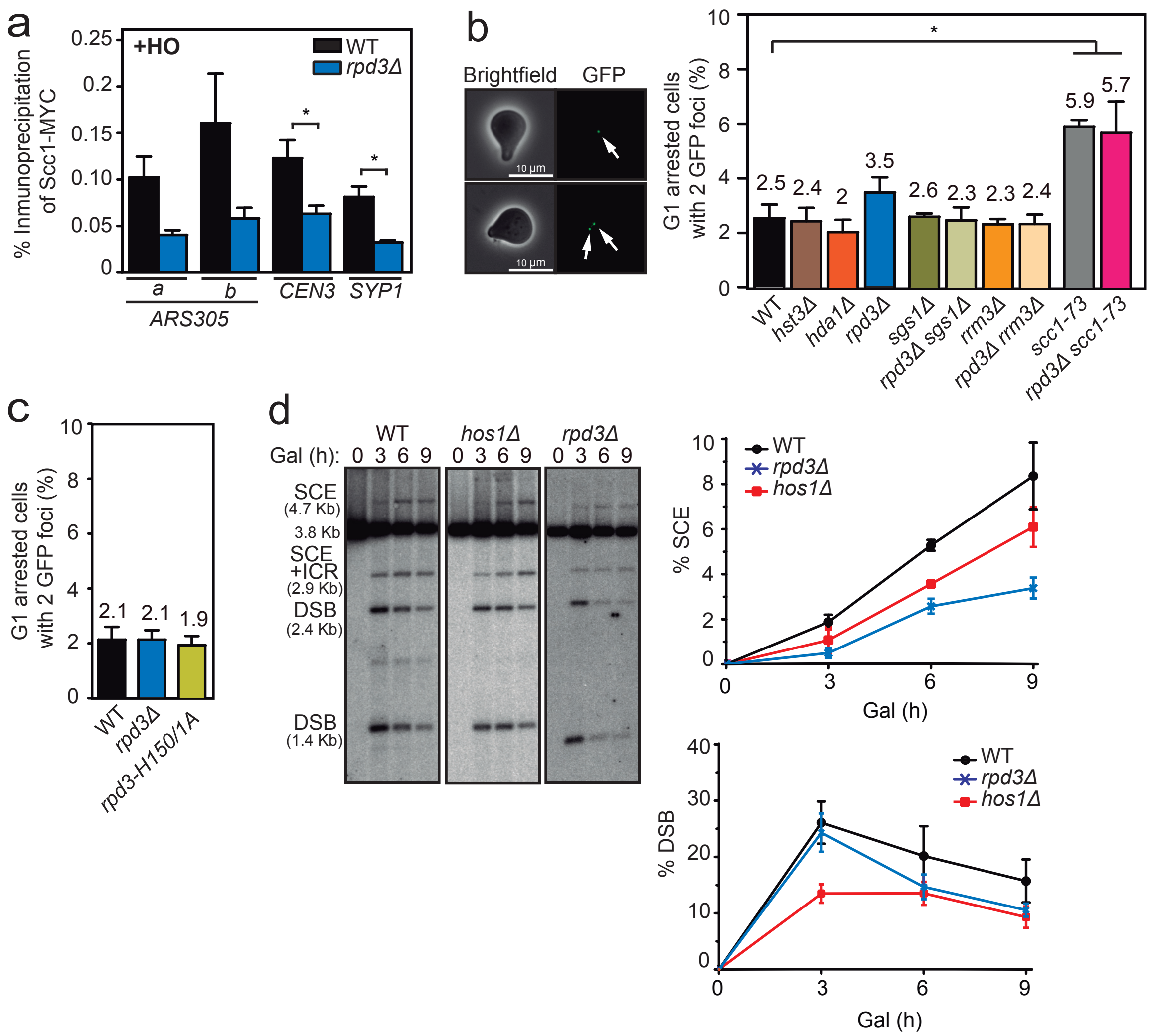

Supplementary Figure 5. Rpd3 and Hda1 favor SCR by promoting cohesin loading and sister chromatid cohesion. Related to Figure 7.

(A) ChIP analysis of Scc1-MYC occupancy after HO-induction in wild-type (WSM) and *rpd3*Δ (WSMR3) strains. A scheme of the different chromosome III regions analysed is depicted on Figure 7 (n = 3).

(B) Percentage of G1 (α-factor) arrested cells that have lost sister chromatid cohesion at chromosome III, as indicated by the appearance of 2 GFP foci, in wild-type (SBY885), *hda1*Δ (SBHDA1), *rpd3*Δ (SBRPD3), *hst3*Δ (SBHST3), *sgs1*Δ (SBSGS1), *rpd3*Δ *sgs1*Δ (SBR3SG), *rrm3*Δ (SBRRM3), *rpd3*Δ *rrm3*Δ (SBR3RR), *scc1-73* (SBSCC1) and *rpd3*Δ *scc1-73* (SBR3S1). Representative images of cells with one or two GFP foci are shown (n ≥ 3).

(C) Percentage of G1 (α-factor) arrested cells that have lost sister chromatid cohesion at chromosome III, as indicated by the appearance of 2 GFP foci, in SBRPD3 (*rpd3*Δ) strain transformed with PEN149 (WT), pRS315 (*rpd3*Δ) or PEN153 (*rpd3-H150/1A*) plasmids (n = 3).

(D) Representative Southern blots and quantification of the 4.7-Kb SCE fragments during a time-course experiment after HO induction performed in wild-type (WSR-7D), *rpd3*Δ (WSRPD3b) and *hos1*Δ (WSHOS1) strains transformed with pRS316-TINV (n ≥ 3). Other details as in Figure 3A.

Means and SEM are plotted in all panels. *p % 0.05; **p % 0.01 (two-tailed Student's t test). In (A), (B) and (C) *p ≤ 0.05 (two-tailed Student's t test). Data underlying this Figure are provided as Source Data file.

Supplementary Table 1.- Strains used in this study.

Name	Genotype	Source
BY4741	*MATa ura3Δ0 leu2Δ0 his3Δ1 met15Δ0*	Euroscarf
Y04276	BY4741 *dot1Δ::KANMX4*	Euroscarf
Y04530	BY4741 *rad54Δ::KANMX4*	Euroscarf
Y04686	BY4741 *rsc1Δ::KANMX4*	Euroscarf
Y05266	BY4741 *rsc2Δ::KANMX4*	Euroscarf
Y02397	BY4741 *arp8Δ::KANMX4*	Euroscarf
Y06502	BY4741 *cac2Δ::KANMX4*	Euroscarf
Y06160	BY4741 *chd1Δ::KANMX4*	Euroscarf
Y00162	BY4741 *chz1Δ::KANMX4*	Euroscarf
Y00389	BY4741 *fun30Δ::KANMX4*	Euroscarf
Y06969	BY4741 *hmo1Δ::KANMX4*	Euroscarf
Y03385	BY4741 *isw1Δ::KANMX4*	Euroscarf
Y01601	BY4741 *isw2Δ::KANMX4*	Euroscarf
Y01586	BY4741 *snf2Δ::KANMX4*	Euroscarf
Y03693	BY4741 *swr1Δ::KANMX4*	Euroscarf
Y07285	BY4741 *gcn5Δ::KANMX4*	Euroscarf
Y05347	BY4741 *hda1Δ::KANMX4*	Euroscarf
Y01114	BY4741 *rpd3Δ::KANMX4*	Euroscarf
Y06209	BY4741 *rco1Δ::KANMX4*	Euroscarf
Y00849	BY4741 *sap30Δ::KANMX4*	Euroscarf
Y03738	BY4741 *sir2Δ::KANMX4*	Euroscarf
Y03171	BY4741 *hmt1Δ::KANMX4*	Euroscarf
Y00186	BY4741 *jhd1Δ::KANMX4*	Euroscarf
Y06922	BY4741 *jhd2Δ::KANMX4*	Euroscarf
Y01257	BY4741 *set2Δ::KANMX4*	Euroscarf
Y03771	BY4741 *bre1Δ::KANMX4*	Euroscarf
Y00809	BY4741 *ubp8Δ::KANMX4*	Euroscarf
WSR-7D	*MATa-inc trp1-1 ura3-1 ade2-1 his3-11,15 can1-100 ade3::gal-HO leu2Δ::SFA1*	1
WSRSC2	WSR-7D *rsc2Δ::KANMX4*	This study
WSFUN30	WSR-7D *fun30Δ::KANMX4*	This study
WSSWR1	WSR-7D *swr1Δ::KANMX4*	This study
WSHDA1	WSR-7D *hda1Δ::KANMX4*	This study
WSRPH1	WSR-7D *rph1Δ::KANMX4*	This study
WSSAP30	WSR-7D *sap30Δ::KANMX4*	This study
WSRPD3	WSR-7D *rpd3Δ::KANMX4*	This study
WSRPD3b	WSR-7D *rpd3Δ::NATNT2*	This study
WSRCO1	WSR-7D *rco1Δ:: NATNT2*	This study
WSSIN3	WSR-7D *sin3Δ:: NATNT2*	This study
WSH1R3	WSR-7D *hda1Δ::NATNT2 rpd3Δ::KANMX4*	This study
W-Lk	*Mata-inc trp1-1 ura3-1 ade2-1 his3-11,15 can1-100 ade3::GAL::HO leu2k::URA-ADE::leu2k*	2
WLHDA1	W-Lk *hda1Δ::KANMX4*	This study

WLSAP30	W-Lk *sap30Δ::KANMX4*	This study
WFLP	*MATa-inc trp1-1 ura3-1 ade2-1 his3-11,15 can1-100 leu2Δ::SFA1 met17Δ::GAL-FLPH305L::HPHMX6*	This study
WFRPD3	WFLP *rpd3Δ::KANMX4*	This study
WFSCC1	WFLP *scc1-73*	This study
WFR3S1	WFLP *scc1-73 rpd3Δ::KANMX4*	This study
USCE	*MATa his3Δ200 ura3-1 lys2-801 his3-Δ3'his3-Δ5':: URA3*	This study
USHDA1	USCE *hda1Δ::KANMX4*	This study
USSAP30	USCE *sap30Δ::KANMX4*	This study
WSRA	*MATa-inc trp1-1 ura3-1 ade2-1 his3-11,15 can1-100 leu2Δ::SFA1*	This study
WRHDA1	WSRA *hda1Δ::KANMX4*	This study
WRSAP30	WSRA *sap30Δ::KANMX4*	This study
WRRAD3	WSRA *rad3-102::HPHMX6*	This study
WRH1RD	WSRA *rad3-102::HPHMX6 hda1Δ::KANMX4*	This study
WRS30RD	WSRA *rad3-102::HPHMX6 sap30Δ::KANMX4*	This study
WS-52	*MATa-inc trp1-1 ura3-1 ade2-1 his3-11,15 can1-100 ade3::gal-HO leu2::SFA1 rad52Δ::KanMX4*	3
JFR-4	*MAT a-inc ade2-1 can1-100 his3-11,15 leu2Δ::SFA1 trp1-1 ura3-1 ade3Δ::GAL-HO GAL::leu2Δ3'::ACT1-iΔ3'::URA3 HYG-HO::ACT1-iΔ5'::leu2Δ5'*	4
JFRPD3	JFR-4 *rpd3Δ::NATNT2*	This study
YKJM1	Mata ura3-52 leu2Δ*1 trp1Δ63 ade2Δ1 ade8 his3Δ200 lys2ΔBgl hom3-100 YEL069::URA3*	5
YKHDA1	YKJM1 *hda1Δ::KANMX4*	This study
YKSAP30	YKJM1 *sap30Δ::KANMX4*	This study
WSRAD51	WSR-7D *rad51Δ::KANMX4*	This study
WSR3R5	WSR-7D *rad51Δ::KANMX4 rpd3Δ::NATNT2*	This study
WSSAE2	WSR-7D *sae2Δ::KANMX4*	This study
WSR3S2	WSR-7D *sae2Δ::KANMX4 rpd3Δ::NATNT2*	This study
WSSGS1	WSR-7D *sgs1Δ::NATNT2*	This study
WSR3SG	WSR-7D *sgs1Δ::KANMX4 rpd3Δ::NATNT2*	This study
WSMUS81	WSR-7D *mus81Δ::KANMX4*	This study
WSR3M8	WSR-7D *mus81Δ::KANMX4 rpd3Δ::NATNT2*	This study
WSRRM3	WSR-7D *rrm3Δ::KANMX4*	This study
WSR3RR	WSR-7D *rrm3Δ::KANMX4 rpd3Δ::NATNT2*	This study
WSHST3	WSR-7D *hst3Δ::KANMX4*	This study
WSR3H3	WSR-7D *hst3Δ::KANMX4 rpd3Δ::NATNT2*	This study
WSSCC1	WSR-7D *scc1-73*	This study
WSR3S1	WSR-7D *scc1-73 rpd3Δ::NATNT2*	This study
WSHOS1	WSR-7D *hos1::KANMX4*	This study
SBY885	*MATa ade2-1 can1-100 bar1-1 leu2-3,112 lys2Δ trp1-1 ura3-1 his3-11::pCUP1-GFP12-LacI12::HIS cenIII-lacO128::TRP1*	Sue Biggins
SBHDA1	SBY885 *hda1Δ::NATNT2*	This study
SBRPD3	SBY885 *rpd3Δ:: KANMX4*	This study

SBHST3	SBY885 *hst3Δ::KANMX4*	This study
SBSGS1	SBY885 *sgs1Δ:: HPHMX6*	This study
SBR3SG	SBY885 *sgs1Δ:: HPHMX6 rpd3Δ::NATNT2*	This study
SBRRM3	SBY885 *rrm3Δ:: HPHMX6*	This study
SBR3RR	SBY885 *rrm3Δ:: HPHMX6 rpd3Δ:: KANMX4*	This study
SBSCC1	SBY885 *scc1-73*	This study
SBR3S1	SBY885 *scc1-73 rpd3Δ:: KANMX4*	This study
WSM	WSR-7D *SCC1-9MYC::HPHNT1*	This study
WSMR3	WSM *rpd3Δ::NATNT2*	This study
WSMH3	WSM *hst3Δ::KANMX4*	This study
WSMRR	WSM *rrm3Δ::KANMX4*	This study

Supplementary Table 2.- Primers used in this study.

Primer name	Sequence
Right-F	TCGAGCGTCCCAAAACCTT
Right-R	CGCGTGTACGCATGTAACATT
Left-F	TGTATTTGTGTTTGCGTGTCTATAGAA
Left-R	TGCATGTGCTCTGTATGTATATAAAACTC
Leu2-1F	GCCCCTAAGAAGATCGTCGTT
Leu2-1R	AATGGCTTCGGCTGTGATTT
Leu2-2F	TGGTGTCGCTTGGGATAGTG
Leu2-2R	CCATGAAAGCGGCCATTCT
Leu2-3F	TGTTGCCATCTGCGTCCTT
Leu2-3R	CGTGGCATGGTTCGTACAAA
Leu2-4F	AGGCCATTGAAGATGCAGTTAAA
Leu2-4R	GGTGGTACTGTTGGAACCACCTA
Cen3-F	GAAACAACAATGGCAAAATATTCG
Cen3-R	CTATATCTGTTGGTTTGTCAAGTTGCT
ARS305a-F	CAAATCTACTTCGGGAGGCAAGT
ARS305a-R	ACCCAGTCTACAATTCATTTCACAGT
ARS305b-F	CCGTCATTGATTAAGTGTCCATTT
ARS305b-R	TTTCCCTGGAAGACGTCATACTC
SYP1-F	CCCGTGGCTTGAGGATTT
SYP1-R	CGCTACCCCTACTTCTTCTTCC
Leu Up 2000	GTTCCACTTCCAGATGAGGC
Leu Lo 2000	TTAGCAAATTGTGGCTTGA
p1	TTCACGCTTACTGCTTTTTTC
p2	CGGAGGCTTCATCGGAG
p3	GCCCTACAACATGAGCCACC
p4	CTAAACATATAATATAGCAACA
*EcoR*I-FRT-1	ATTGGCAAGCGAATTCAGAAGTTCCTATACTTTCTAGAGAA TAGGAACTTCCGAATAGGAACTTCGAATTCAGGTCGATAA
*EcoR*I-FRT-2	TTATCGACCTGAATTCGAAGTTCCTATTCGGAAGTTCCTA TTCTCTAGAAAGTATAGGAACTTCTGAATTCGCTTGCCAAT

Supplementary References

1 Munoz-Galvan, S., Jimeno, S., Rothstein, R. & Aguilera, A. Histone H3K56 acetylation, Rad52, and non-DNA repair factors control double-strand break repair choice with the sister chromatid. *PLoS genetics* **9**, e1003237, doi:10.1371/journal.pgen.1003237 (2013).

2 Munoz-Galvan, S. *et al.* Competing roles of DNA end resection and non-homologous end joining functions in the repair of replication-born double-strand breaks by sister-chromatid recombination. *Nucleic Acids Res* **41**, 1669-1683, doi:10.1093/nar/gks1274 (2013).

3 Gonzalez-Barrera, S., Cortes-Ledesma, F., Wellinger, R. E. & Aguilera, A. Equal sister chromatid exchange is a major mechanism of double-strand break repair in yeast. *Mol Cell* **11**, 1661-1671 (2003).

4 Ruiz, J. F., Gomez-Gonzalez, B. & Aguilera, A. Chromosomal translocations caused by either pol32-dependent or pol32-independent triparental break-induced replication. *Mol Cell Biol* **29**, 5441-5454 (2009).

5 Schmidt, K. H., Pennaneach, V., Putnam, C. D. & Kolodner, R. D. Analysis of gross-chromosomal rearrangements in Saccharomyces cerevisiae. *Methods in enzymology* **409**, 462-476 (2006).
